# Finite Element Method-Based Dynamic Response of Micropolar Polymers with Voids

**DOI:** 10.3390/polym13213727

**Published:** 2021-10-28

**Authors:** Sorin Vlase, Marin Marin

**Affiliations:** 1 Department of Mechanical Engineering, Transilvania University of Brașov, B-dul Eroilor 20, 500036 Brașov, Romania; 2 Romanian Academy of Technical Sciences, B-dul Dacia 26, 030167 Bucharest, Romania; 3 Department of Mathematics, Transilvania University of Brașov, B-dul Eroilor 20, 500036 Brașov, Romania

**Keywords:** micropolar, voids, finite element, minimum principle, Lagrange

## Abstract

Composite-based polymer materials are manufactured in a wide variety of types with different compositions, structures, geometries, and topological descriptions. Among these, micropolar materials with voids have become increasingly studied in the literature. This paper establishes the equations of motion for such a material for the purpose of dynamic analysis via the finite element method (FEM). The Euler–Lagrangian formalism, based on the expressions of kinetic energy, potential energy, and mechanical work, is used. Hence, it is possible to study the dynamic response of such a system in the most general configuration case. The choice of the shape functions will determine the matrix coefficients for each particular case. An application illustrates the presented results.

## 1. Introduction

Among the new high-performance materials and among the polymers used in industry and in a number of practical applications, there are some that may have voids due to the manufacturing process or phenomena that occur during their use. As a result, researchers’ attention has increasingly begun to turn to this type of material, both theoretically and experimentally.

The need for and advantages of void structures have been emphasized in reference works [1], and the voids, their distribution, and their geometry are presented in [2,3]. The appearance of voids in different polymeric materials is studied by researchers, because in a number of cases and technical applications, the formation of these voids is undesirable and their production must be controlled. Presented in [4] is the generation of submicrometer voids within a PMMA polymer. These voids can be organized in a multilayer structure and can be used practically for optical storage of high-density data. In [5] we studied the appearance of these voids in the case of adhesive bonding and how their existence influences the properties of the material obtained. In thin polymer films, coagulated from isotropic solutions, voids with a size of the order of 100 μm are often found. In order to control the mechanism of the formation of these types of voids so that, in the end, the quality of the film is as high as possible, studies of such materials were made. The results obtained in [6] present some useful conclusions for manufacturers regarding the formation of these large voids. The potential of using void materials in practical applications is presented in papers, such as [7,8]. Voids can not only be harmful and, therefore, undesirable in the case of their appearance in materials, but in certain cases and circumstances, they can also influence the physical properties of the materials, so they can be used in specific practical applications.

Theoretical models for studying the behavior of polymers with voids have been used by researchers [9,10,11] to determine the physical properties of various materials used in engineering. These works are useful for designers because they give information about the physical properties of these materials. It is noted that the models presented are generally analytical models. The element method was less used in this case, especially due to the complexity of the necessary operations [12].

More numerous studies have been devoted to obtaining data on the properties of voids polymers using experimental methods. In this way, it was possible to determine different engineering constants necessary for the design of systems that use polymeric materials [13,14,15,16].

The finite element method (FEM) has recently been intensively developed, being able to analyze mechanical structures from different material, namely, isotropic, transversely isotropic or anisotropic, composite, sandwich type, or under special conditions (for example, under the action of a field of temperature) [17,18,19]. In all of these cases, the method proved to be very useful and a powerful analysis tool [20,21].

Methods for writing equations and different application situations have been developed [22,23].

The use of the finite element method in the study of composite materials has existed for a long time [24,25,26,27,28], and, at the moment, in the bank of elements of known software, there are also different types of composite material.

At present, for the materials currently used in practical applications, there are numerous studies that deal with the determination of the physical properties of composite, bi-, or multi-phase materials [29,30,31,32]. New research directions are also developing that lead to interesting results and the extension of the field in which composite materials can be applied [33,34,35,36].

The first works to study the properties of bodies with voids are due to [37]. The main idea introduced was to consider an additional degree of kinematic freedom. Based on this new parameter, a theory has been developed to describe the behavior of flowing granular materials. In [38], a theoretically substantiated study of elastic materials with voids was presented. The author of [39] developed and extended the theory considering the temperature by establishing the thermoelasticity equations of materials with voids, and in [40], the author moved to a new level and applied the theory of semigroups in this field, demonstrating the existence and uniqueness of solutions. The aim of this paper is to use the results obtained in the development of this theory, using numerical procedures, in our case, the FEM. Mechanics of the bodies with voids are useful for the study of a large number of practical applications in solid mechanics [41]. The main application of these methods is for the manufactured porous materials, but also for other fields, such as the study of geological materials (rocks and soils) [42].

Numerous calculation methods are used for the analysis of mechanical systems, essentially equivalent in terms of the results obtained. For complex systems, analytical mechanics is, in general, the best analysis tool. In the study of an elastic solid using the FEM, analytical methods remain the best option, allowing for a systematic and orderly approach. Analytical mechanics enable (starting from the well-known axioms of mechanics), through a collection of alternative mathematical formulations, an adequate mathematical description to be obtained, which is useful for numerical approaches.

The constraints that appear in an FEM model, which are due to the connection between the elements, significantly decrease the number of degrees of freedom (DOF), and analytical mechanics becomes the best method to describe the system. The formalism offered by analytical mechanics has the great advantage of being a simple representation of the equations and offering the possibility to easily apply the classical algorithms of the FEM.

In the manufacture of composite materials, a void is a pore that remains usually occupied by air, not being filled with polymer or fiber. Voids therefore represent a defect that occurs in the manufacturing process and are generally undesirable. This is because they can affect the physical properties of the material and, therefore, its strength, which is often the main quality that a composite must have. In addition, voids can act as places for the initiation of cracks, so they can significantly influence the lifespan. Moreover, they can allow moisture to enter the material and change the isotropy of the material. In engineering, a void content of 3–5% may be acceptable, but in the aerospace industry, it is obvious that this level must be lower.

We mention some papers that study, using the FEM, the micropolar body with voids. However, the indicated papers studied only the elastostatic cases and not the dynamic response of such a system [43,44,45]. From this point of view, this paper can be considered original.

Voids are essentially a manufacturing defect, which manifests itself especially when the density of fibers in the case of composite materials is high. In this case, it is difficult for resin to occupy all of the air spaces that exist between the fibers used. Experimentally, it has been found that voids have an influence that cannot be neglected, in the sense of decreasing the mechanical properties of the material in which they appear, so it is necessary to pay more attention to design if it is known that such materials will be used. There are serious difficulties in suppressing voids that occur during manufacturing. Therefore, it is difficult to impose the realization of materials without voids and, as such, it is necessary to know their influence on mechanical properties, and to establish how acceptable their existence in a polymeric material can be and what the acceptable percentage of voids must be.

The paper aims to determine the equations of motion, using the FEM, in order to determine the complex behavior of a composite material with voids. In the paper, we introduce some basic notion in the FEM, written for this special material with voids and basic notation in kinematics of the elastic bodies, useful in the following consideration. Next, we compute the kinetic energy, potential energy, and work of different types of applied forces for a micropolar body with voids [46,47]. Then, we apply the Lagrange equations in order to obtain the second-order differential equations for a three-dimensional finite element, which is the goal of the paper.

## 2. Notation, Formulas, and Methods

In the following, the notation used in [37,38,39,40,41,42] is introduced.

The mass density ρ(x,y,z) of a body with voids is expressed by:

(1) ρ(x,y,z)=v(x,y,z) γ(x,y,z).

Here, γ(x,y,z) is the mass density (without voids); v(x,y,z) represents the volume fraction of material (0<ν≤1) and is a measure in the volume change of original material resulting from void distension or compaction.

If we consider the reference configuration, the mass density is expressed by:

(2) $$\rho_{o}(x,y,z)=v_{o}(x,y,z)\gamma_{o}(x,y,z).$$

In our study, a fixed reference frame is considered. An arbitrary point *M* in the domain *B* is denoted by (*x*,*y*,*z*) and in *B_o_* by (*x_o_*,*y_o_*,*z_o_*). Time is measured by the variable *t*, where $t\in \left[0,t_{o}\right.)$. In the following, the Latin indices range over the integers (1, 2, 3) and the Greek indices have the range (1, 2).

The FEM is a powerful tool that can be used to study the mechanical response of composite materials [48,49,50,51,52,53]. The method used in many engineering applications to obtain the motion equations for different types of materials is Lagrange’s equation. Firstly, it is necessary to obtain the response for a single finite element. We consider, in this study, the shape function arbitrarily chosen at this level. The relations will be obtained in the local system of coordinates, but it is possible to transfer them to a global reference frame, using an appropriate transformation matrix. The next step is the assembling of the obtained equations systems to determine the dynamic response of the modeled structure. Introducing the boundary conditions and the external applied loads over the elastic system, it is possible to obtain the solution to the differential equations. As a hypothesis, the deformations can be considered small enough so that the general “rigid body motion” of the mechanical system is not influenced by these deformations.

Most papers that establish the equations of the evolution of a finite element being in rigid motion use the method of Lagrange’s equation to obtain the system of differential equations. The method proved to be useful and relatively simple to apply. The use of relatively simple and well-known notions from classical mechanics, such as kinetic energy, potential energy, and mechanical work, is an advantage of the method. According to the authors, there is no similar work addressing the use of the FEM in the analysis of polymeric materials with voids. The paper aims to provide a formalism in the field that will allow further developments on multiple levels on materials with voids, the use of different types of finite elements, and the application of other methods used by analytical mechanics to obtain equations of motion.

The fact that the liaison forces are not involved in the final form of the equations of motion is another important advantage. This becomes particularly important in the FEM, where modeled systems have a very large number of DOFs when the application of this method leads to a significant decrease in the number of unknowns and, consequently, in the number of calculations, becoming useful for users. However, alternative methods can be used to write equations of motion, for example, the equations of Hamilton, Maggi, or Gibbs–Appell, but only time and subsequent applications will ultimately decide which method is most advantageous for such an approach.

## 3. Kinematics, Kinetic Energy, Potential Energy, and Work

### 3.1. Kinematics

The basis of the FEM is to consider the displacement of a certain point of an elastic body defined by shape (interpolation) functions expressed using independent coordinates from some specific points of the rigid called nodes. These independent coordinates can be considered the coordinate points, some of their derivatives, or other significant kinematic elements.

We denote with $\bar{\omega }$ the angular velocity, with $\bar{\varepsilon }$ the angular acceleration of the finite element considered, with ${\bar{v}}_{o}$ the velocity, and with ${\bar{a}}_{o}$ the acceleration of the origin $O(X_{o},Y_{o},Z_{o})$ of the mobile coordinate frame.

We use the indices *L* for local and G for global reference frames to express some sizes in the corresponding reference frame. The non-indexed vectors and matrices are considered in the local coordinate system. The orthonormal operator $\left[R\right]$ changes a vector $\left\{t\right\}$ from the local to the global reference frame:

(3) $${\left\{t\right\}}_{G}=\left[R\right]{\left\{t\right\}}_{L}.$$

The point *M* belonging to the elastic finite element becomes, after a short time interval, *M*′:

(4) $${\left\{r_{M\prime }\right\}}_{G}={\left\{r_{O}\right\}}_{G}+\left[R\right]\left({\left\{r\right\}}_{L}+{\left\{u\right\}}_{L}\right).$$

where ${\left\{r_{M}\right\}}_{G}$ is the position vector of the point *M*, ${\left\{r_{M\prime }\right\}}_{G}$ of the point *M*′, ${\left\{u\right\}}_{L}$ represents the displacement vector, ${\left\{\varphi \right\}}_{L}$ the vector of rotations, and ${\left\{r_{O}\right\}}_{G}$ the position vector of the point *O*.

In the FEM, the continuous vector field of the displacements is expressed by:

(5) $${\left\{u\right\}}_{L}=\left[N_{\delta }\right]{\left\{\delta \right\}}_{L};\;{\left\{\varphi \right\}}_{L}=[N_{\varphi }]{\left\{\delta \right\}}_{L}$$

where ${\left\{\delta \right\}}_{L}$ is the vector of nodal independent coordinates usually used in the FEM and $\left[N_{\delta }\right]$ and $\left[N_{\varphi }\right]$ are shape function matrices. The explicit components of this vector depend, in each case, on the type of finite element chosen; in the case of an application, it will be made explicit by the user for each case [54]. Differentiating Equation (4), the velocity of *M*′ is obtained:

(6) $${\left\{v_{M\prime }\right\}}_{G}={\left\{{\dot{r}}_{M\prime }\right\}}_{G}={\left\{{\dot{r}}_{O}\right\}}_{G}+\left[\dot{R}\right]\left({\left\{r\right\}}_{L}+\left[N\right]{\left\{\delta \right\}}_{L}\right)+\left[R\right]\left[N\right]{\left\{\dot{\delta }\right\}}_{L}.$$

In the local coordinate frame, the velocity becomes:

(7) $${\left\{v_{M\prime }\right\}}_{L}={\left[R\right]}^{T}{\left\{v_{M\prime }\right\}}_{G}={\left\{{\dot{r}}_{O}\right\}}_{L}+{\left[R\right]}^{T}\left[\dot{R}\right]\left({\left\{r\right\}}_{L}+\left[N_{\delta }\right]{\left\{\delta \right\}}_{L}\right)+\left[N_{\delta }\right]\;{\left\{\dot{\delta }\right\}}_{L}.$$

We denote:

(8) $$\left[A\right]={\left[R\right]}^{T}\left[\dot{R}\right];\;\left[B\right]={\left[R\right]}^{T}\left[\dot{R}\right]\left[N_{\delta }\right];\;\left[C\right]=\left[N_{\delta }\right];\;\left[D\right]=\left[N_{\varphi }\right].$$

Using this notation, the expression for the velocity of an arbitrary point of the body is:

(9) $${\left\{v_{M\prime }\right\}}_{L}=\left[\begin{array}{cccc} E_{3} & \left[A\right] & \left[B\right] & \;\left[C\right] \end{array}\right]\left[\begin{array}{c} {\left\{{\dot{r}}_{O}\right\}}_{L}\\ {\left\{r\right\}}_{L}\\ {\left\{\delta \right\}}_{L}\\ {\left\{\dot{\delta }\right\}}_{L} \end{array}\right].$$

Differentiating Equation (5), it is possible to obtain the dependence of the nodal angular velocity as:

(10) $${\left\{\dot{\varphi }\right\}}_{L}=\left[N_{\varphi }\right]{\left\{\dot{\delta }\right\}}_{L}=\left[D\right]{\left\{\dot{\delta }\right\}}_{L}$$

(11) $${\left\{\dot{\nu }\right\}}_{L}=\left[N_{\nu }\right]{\left\{\dot{\delta }\right\}}_{L}$$

### 3.2. Kinetic Energy

Having Equations (9) and (10), it is now possible to obtain the expression of kinetic energy of the finite element being in motion, considered as a material with voids:

(12) $$E_{c}=\frac{1}{2}\int_{V}\rho_{0}{\left\{v_{M\prime }\right\}}_{G}^{T}{\left\{v_{M\prime }\right\}}_{G}dV+\frac{1}{2}\int_{V}\rho_{0}{\left\{{\dot{\varphi }}_{M\prime }\right\}}_{G}^{T}\left[Y\right]{\left\{{\dot{\varphi }}_{M\prime }\right\}}_{G}dV+\frac{1}{2}\int_{V}\rho_{0}\kappa_{\nu }{\dot{\nu }}_{M\prime }^{2}{}_{G}dV.$$

where κ is the equilibrated inertia and $\left[Y\right]$ the matrix containing the coefficients of inertia [38,39,40,41]. The classic transformation relations between vectors is written in the two reference frames:

(13) $${\left\{\delta \right\}}_{G}=\left[R\right]{\left\{\delta \right\}}_{L};\;{\left\{\varphi \right\}}_{G}=\left[R\right]{\left\{\varphi \right\}}_{L}$$

and

(14) $${\left\{\delta \right\}}_{L}={\left[R\right]}^{T}{\left\{\delta \right\}}_{G};\;{\left\{\varphi \right\}}_{L}={\left[R\right]}^{T}{\left\{\varphi \right\}}_{G}$$

which make it possible to obtain, for kinetic energy, expressed in the local reference frame, the form:

(15) $$\begin{array}{l} E_{C}=\frac{1}{2}\int_{V}\left[\begin{array}{cccc} {\left\{{\dot{r}}_{O}\right\}}_{L}^{T} & {\left\{r\right\}}_{L}^{T} & {\left\{\delta \right\}}_{L}^{T} & {\left\{\dot{\delta }\right\}}_{L}^{T}\;{\left\{\dot{\varphi }\right\}}_{L}^{T}\;{\dot{\nu }}_{M'} \end{array}\right]x\\ \left[\begin{array}{cccccc} E_{3} & \left[A\right] & \left[B\right] & \left[C\right] & 0 & 0\\ {\left[A\right]}^{T} & {\left[A\right]}^{T}\left[A\right] & {\left[A\right]}^{T}\left[B\right] & {\left[A\right]}^{T}\left[C\right] & 0 & 0\\ {\left[B\right]}^{T} & {\left[B\right]}^{T}\left[A\right] & {\left[B\right]}^{T}\left[B\right] & {\left[B\right]}^{T}\left[C\right] & 0 & 0\\ {\left[C\right]}^{T} & {\left[C\right]}^{T}\left[A\right] & {\left[C\right]}^{T}\left[B\right] & {\left[C\right]}^{T}\left[C\right] & 0 & 0\\ 0 & 0 & 0 & 0 & \left[Y\right] & 0\\ 0 & 0 & 0 & 0 & 0 & \kappa_{v} \end{array}\right]\left\{\begin{array}{c} {\left\{{\dot{r}}_{O}\right\}}_{L}\\ {\left\{r\right\}}_{L}\\ {\left\{\delta \right\}}_{L}\\ {\left\{\dot{\delta }\right\}}_{L}\\ {\left\{\dot{\varphi }\right\}}_{L}\\ {\dot{\nu }}_{M'} \end{array}\right\}\rho_{o}dV \end{array}$$

This term is used in Lagrange’s equations. From the 12 terms that appear by developing this expression of energy, only 7 are useful in Lagrange’s equations, the derivatives of the others being equal to zero. We highlight in the following the terms that matter in Lagrange’s equations:

(16) $$E_{C\delta }={\left\{\delta \right\}}_{L}^{T}\left[\left(\int_{V}{\left[N_{\delta }\right]}^{T}{\left[\dot{R}\right]}^{T}\left[R\right]\rho_{o}dV\right){\left\{{\dot{r}}_{O}\right\}}_{L}+\left(\int_{V}{\left[N_{\delta }\right]}^{T}{\left[R\right]}^{T}\left[\dot{R}\right]{\left\{r\right\}}_{L}\rho_{o}dV\right)\right];$$

(17) $$E_{C\delta \delta }=\frac{1}{2}{\left\{\delta \right\}}_{L}^{T}\left(\int_{V}{\left[N_{\delta }\right]}^{T}{\left[\dot{R}\right]}^{T}\left[\dot{R}\right]\left[N_{\delta }\right]\rho_{o}dV\right){\left\{\delta \right\}}_{L};$$

(18) $$E_{C\delta \dot{\delta }}={\left\{\delta \right\}}_{L}^{T}\left(\int_{V}{\left[N_{\delta }\right]}^{T}{\left[\dot{R}\right]}^{T}\left[R\right]\left[N_{\delta }\right]\rho_{o}dV\right){\left\{\dot{\delta }\right\}}_{L};$$

(19) $$E_{C\dot{\delta }}={\left\{\dot{\delta }\right\}}_{L}^{T}\left[\left(\int_{V}{\left[N_{\delta }\right]}^{T}\rho_{o}dV\right){\left\{{\dot{r}}_{O}\right\}}_{L}+\left(\int_{V}{\left[N_{\delta }\right]}^{T}{\left[R\right]}^{T}\left[\dot{R}\right]{\left\{r\right\}}_{L}\rho_{o}dV\right)\right];$$

(20) $$E_{C\dot{\delta }\dot{\delta }}=\frac{1}{2}{\left\{\dot{\delta }\right\}}_{L}^{T}\left(\int_{V}{\left[N_{\delta }\right]}^{T}\left[N_{\delta }\right]\rho_{o}dV\right){\left\{\dot{\delta }\right\}}_{L};$$

(21) $$E_{C\varphi }=\frac{1}{2}{\left\{\dot{\delta }\right\}}_{L}^{T}\left(\int_{V}{\left[N_{\varphi }\right]}^{T}\left[Y\right]\left[N_{\varphi }\right]\rho_{o}dV\right){\left\{\dot{\delta }\right\}}_{L};$$

(22) $$E_{C\nu }=\frac{1}{2}{\left\{\dot{\delta }\right\}}_{L}^{T}\left(\int_{V}{\left[N_{\varphi }\right]}^{T}\left[Y\right]\left[N_{\varphi }\right]\rho_{o}dV\right){\left\{\dot{\delta }\right\}}_{L}.$$

### 3.3. Internal Energy

The expression of the potential energy is:

(23) $$E_{p}=\frac{1}{2}\int_{V}{\left\{\sigma \right\}}^{T}\left\{\varepsilon \right\}dV.$$

where $\left\{\varepsilon \right\}$ is the strain vector with dimensions *9 × 1*, corresponding to the strain tensor $\left[\varepsilon \right]$ tensor, and $\left\{\sigma \right\}$ the stress vector is obtained in a similar way.

Using the Hooke law:

(24) $$\left\{\sigma \right\}=\left[H\right]\left\{\varepsilon \right\}.$$

and the relationships between strains and finite deformations:

(25) $$\left\{\varepsilon \right\}=\left[b_{1}\right]\left\{u\right\}+\left[b_{2}\right]\left\{\varphi \right\}=\left[b_{1}\right]\left[N_{\delta }\right]{\left\{\delta \right\}}_{L}+\left[b_{2}\right]\left[N_{\varphi }\right]{\left\{\delta \right\}}_{L}=\left(\left[b_{1}\right]\left[N_{\delta }\right]+\left[b_{2}\right]\left[N_{\varphi }\right]\right){\left\{\delta \right\}}_{L}=\left[N*\right]{\left\{\delta \right\}}_{L}.$$

where $\left[b_{1}\right]$ and $\left[b_{2}\right]$ are two differentiation operators (see [54]), Equation (11) becomes:

(26) $$E_{p}=\frac{1}{2}{\left\{\delta \right\}}_{L}^{T}\left(\int_{V}{\left[N*\right]}^{T}{\left[H\right]}^{T}\left[N*\right]dV\right){\left\{\delta \right\}}_{L}.$$

If $\left[k\right]$ denotes the classic rigidity matrix of the material, then:

(27) $$\left[k\right]=\int_{V}{\left[N*\right]}^{T}{\left[H\right]}^{T}\left[N*\right]dV.$$

where:

(28) $$\left[N*\right]=\left[b_{1}\right]\left[N_{\delta }\right]+\left[b_{2}\right]\left[N_{\varphi }\right].$$

Considering Equations (15) and (16), Equation (14) becomes:

(29) $$E_{p}=\frac{1}{2}{\left\{\delta \right\}}_{L}^{T}\left[k\right]{\left\{\delta \right\}}_{L}.$$

At the same time, there exists a term due to the voids, presented in [26], that can be written as:

(30) $$E_{pv}=\frac{1}{2}{\left\{\delta \right\}}_{L}^{T}\left[k_{v}\right]{\left\{\delta \right\}}_{L}.$$

### 3.4. Work

Two types of external loads will be considered: the concentrated forces and moments in knots and volume (distributed) forces $\left\{p\right\}=\left\{p(x,y,z)\right\}$ and moments $\left\{m\right\}=\left\{m(x,y,z)\right\}$. The concentrated forces and moments give a mechanical work:

(31) $$W^{c}={\left\{q_{t}\right\}}_{L}^{T}{\left\{\delta \right\}}_{L}+{\left\{q_{r}\right\}}_{L}^{T}{\left\{\varphi \right\}}_{L}={\left\{q\right\}}_{L}^{T}{\left\{\delta \right\}}_{L}.$$

and the vectors of volume forces and moments give the work:

(32) $$\begin{array}{l} W^{d}=\int_{V}{\left\{p\right\}}_{L}^{T}{\left\{u\right\}}_{L}dV+\int_{V}{\left\{m\right\}}_{L}^{T}{\left\{\varphi \right\}}_{L}dV\\ =\left(\int_{V}{\left\{p\right\}}_{L}^{T}\left[N_{\delta }\right]dV\right){\left\{\delta \right\}}_{L}+\left(\int_{V}{\left\{m\right\}}_{L}^{T}\left[N_{\varphi }\right]dV\right){\left\{\delta \right\}}_{L}={\left\{q^{*}\right\}}_{L}^{T}{\left\{\delta \right\}}_{L} \end{array}$$

The work due to the existence of voids can be expressed as:

(33) $$W^{v}=\int_{V}L\nu dV=\left(\int_{V}L\left[N_{\nu }\right]dV\right){\left\{\delta \right\}}_{L}={\left\{q_{v}\right\}}^{T}{\left\{\delta \right\}}_{L}.$$

### 3.5. Lagrangian

The Lagrangian is [21]:

(34) $$L=E_{c}-E_{p}+W^{d}+W^{c}+W^{v}.$$

Using Equations (15) and (18)–(20), we obtain:

(35) $$\begin{array}{l} L=E_{c}=\frac{1}{2}\int_{V}\rho_{0}{\left\{{\dot{x}}_{M\prime }\right\}}_{G}^{T}{\left\{{\dot{x}}_{M\prime }\right\}}_{G}dV+\frac{1}{2}\int_{V}\rho_{0}{\left\{{\dot{\varphi }}_{M\prime }\right\}}_{G}^{T}\left[Y\right]{\left\{{\dot{\varphi }}_{M\prime }\right\}}_{G}dV+\frac{1}{2}\int_{V}\rho_{0}\kappa {\dot{\nu }}_{M\prime }^{2}{}_{G}dV\;V+\\ -\frac{1}{2}{\left\{\delta \right\}}_{L}^{T}\left[k\right]\left\{\delta \right\}-\frac{1}{2}{\left\{\delta \right\}}_{L}^{T}\left[k_{v}\right]\left\{\delta \right\}+{\left\{q\right\}}_{L}^{T}{\left\{\delta \right\}}_{L}+{\left\{q^{*}\right\}}_{L}^{T}{\left\{\delta \right\}}_{L}+{\left\{q_{v}\right\}}^{T}{\left\{\delta \right\}}_{L}. \end{array}$$

## 4. Euler–Lagrange Equations

The Euler–Lagrange equations are:

(36) $$\frac{d}{dt}{\left\{\frac{\partial L}{\partial \dot{\delta }}\right\}}_{L}-{\left\{\frac{\partial L}{\partial \delta }\right\}}_{L}=0.$$

The term $\left\{\frac{\partial E}{\partial X}\right\}$ denotes the expression:

(37) $$\left\{\frac{\partial E}{\partial X}\right\}=\left\{\begin{array}{c} \frac{\partial E}{\partial x_{1}}\\ \frac{\partial E}{\partial x_{2}}\\ \vdots \\ \frac{\partial E}{\partial x_{n}} \end{array}\right\}.$$

where *E* is a scalar quantity and *X* is the vector $\left\{X\right\}={\left[x_{1}\;x_{2}\;\ldots \ldots \;x_{n}\right]}^{T}$. The following calculus offers us the terms of the motion equations:

(38) $${\left\{\frac{\partial E_{C\delta \dot{\delta }}}{\partial \dot{\delta }}\right\}}_{L}=\left(\int_{V}{\left[N_{\delta }\right]}^{T}{\left[\dot{R}\right]}^{T}\left[R\right]\left[N_{\delta }\right]\rho_{o}dV\right){\left\{\delta \right\}}_{L}.$$

(39) $${\left\{\frac{\partial E_{C\dot{\delta }}}{\partial \dot{\delta }}\right\}}_{L}=\left[\left(\int_{V}{\left[N_{\delta }\right]}^{T}\rho_{o}dV\right){\left\{{\dot{r}}_{O}\right\}}_{L}+\left(\int_{V}{\left[N_{\delta }\right]}^{T}{\left[R\right]}^{T}\left[\dot{R}\right]{\left\{r\right\}}_{L}\rho_{o}dV\right)\right].$$

(40) $${\left\{\frac{\partial E_{C\dot{\delta }\dot{\delta }}}{\partial \dot{\delta }}\right\}}_{L}=\left(\int_{V}{\left[N_{\delta }\right]}^{T}\left[N_{\delta }\right]\rho_{o}dV\right){\left\{\dot{\delta }\right\}}_{L}.$$

(41) $${\left\{\frac{\partial E_{C\varphi }}{\partial \dot{\delta }}\right\}}_{L}=\left(\int_{V}{\left[N_{\varphi }\right]}^{T}\left[Y\right]\left[N_{\varphi }\right]\rho_{o}dV\right){\left\{\dot{\delta }\right\}}_{L}.$$

(42) $$\left\{\frac{\partial E_{C\nu }}{\partial \dot{\delta }}\right\}=\frac{1}{2}{\left\{\dot{\delta }\right\}}_{L}^{T}\left(\int_{V}{\left[N_{\varphi }\right]}^{T}\left[Y\right]\left[N_{\varphi }\right]\rho_{o}dV\right){\left\{\dot{\delta }\right\}}_{L}.$$

(43) $$\frac{d}{dt}{\left\{\frac{\partial E_{C\delta \dot{\delta }}}{\partial \dot{\delta }}\right\}}_{L}=\left(\int_{V}{\left[N_{\delta }\right]}^{T}{\left[\dot{R}\right]}^{T}\left[R\right]\left[N_{\delta }\right]\rho_{o}dV\right){\left\{\dot{\delta }\right\}}_{L}+\left(\int_{V}{\left[N_{\delta }\right]}^{T}\left({\left[\ddot{R}\right]}^{T}\left[R\right]+{\left[\dot{R}\right]}^{T}\left[\dot{R}\right]\right)\left[N_{\delta }\right]\rho_{o}dV\right){\left\{\delta \right\}}_{L}$$

(44) $$\frac{d}{dt}{\left\{\frac{\partial E_{C\dot{\delta }}}{\partial \dot{\delta }}\right\}}_{L}=\left[\left(\int_{V}{\left[N_{\delta }\right]}^{T}\rho_{o}dV\right){\left\{{\ddot{r}}_{O}\right\}}_{L}+\left(\int_{V}{\left[N_{\delta }\right]}^{T}\left({\left[\dot{R}\right]}^{T}\left[\dot{R}\right]+{\left[R\right]}^{T}\left[\ddot{R}\right]\right){\left\{r\right\}}_{L}\rho_{o}dV\right)\right].$$

(45) $$\frac{d}{dt}{\left\{\frac{\partial E_{C\dot{\delta }\dot{\delta }}}{\partial \dot{\delta }}\right\}}_{L}=\left(\int_{V}{\left[N_{\delta }\right]}^{T}\left[N_{\delta }\right]\rho_{o}dV\right){\left\{\ddot{\delta }\right\}}_{L}.$$

(46) $$\frac{d}{dt}\left\{\frac{\partial E_{C\varphi }}{\partial {\dot{\delta }}_{L}}\right\}=\left(\int_{V}{\left[N_{\varphi }\right]}^{T}\left[Y\right]\left[N_{\varphi }\right]\rho_{o}dV\right){\left\{\ddot{\delta }\right\}}_{L}.$$

(47) $$\frac{d}{dt}\left\{\frac{\partial E_{C\nu }}{\partial \dot{\delta }}\right\}=\left(\int_{V}{\left[N_{\nu }\right]}^{T}\left[N_{\nu }\right]\kappa \rho_{o}dV\right){\left\{\ddot{\delta }\right\}}_{L}.$$

(48) $$-{\left\{\frac{\partial E_{C\delta }}{\partial \delta }\right\}}_{L}=-\left(\int_{V}{\left[N_{\delta }\right]}^{T}{\left[\dot{R}\right]}^{T}\left[R\right]\rho_{o}dV\right){\left\{{\dot{r}}_{O}\right\}}_{L}-\left(\int_{V}{\left[N_{\delta }\right]}^{T}{\left[R\right]}^{T}\left[\dot{R}\right]{\left\{r\right\}}_{L}\rho_{o}dV\right).$$

(49) $$-{\left\{\frac{\partial E_{C\delta \delta }}{\partial \delta }\right\}}_{L}=-\left(\int_{V}{\left[N_{\delta }\right]}^{T}{\left[\dot{R}\right]}^{T}\left[\dot{R}\right]\left[N_{\delta }\right]\rho_{o}dV\right){\left\{\delta \right\}}_{L}.$$

(50) $$-{\left\{\frac{\partial E_{C\delta \dot{\delta }}}{\partial \delta }\right\}}_{L}=-\left(\int_{V}{\left[N_{\delta }\right]}^{T}{\left[\dot{R}\right]}^{T}\left[R\right]\left[N_{\delta }\right]\rho_{o}dV\right){\left\{\dot{\delta }\right\}}_{L}.$$

(51) $$-\left\{\frac{\partial \left(-E_{p}+W^{d}+W^{c}+W^{v}\right)}{\partial \delta_{L}}\right\}=\left[k\right]{\left\{\delta \right\}}_{L}+\left[k_{v}\right]{\left\{\delta \right\}}_{L}-{\left\{q\right\}}_{L}^{T}-{\left\{q^{*}\right\}}_{L}^{T}-{\left\{q_{v}\right\}}^{T}.$$

Adding all the terms according to Lagrange’s equations, we obtain:

(52) $$\begin{array}{c} \left[\left(\int_{V}{\left[N_{\delta }\right]}^{T}\left[N_{\delta }\right]\rho_{o}dV\right)+\left(\int_{V}{\left[N_{\varphi }\right]}^{T}\left[Y\right]\left[N_{\varphi }\right]\rho_{o}dV\right)+\left(\int_{V}{\left[N_{\nu }\right]}^{T}\left[N_{\nu }\right]\kappa \rho_{o}dV\right)\right]{\left\{\ddot{\delta }\right\}}_{L}.\\ +2\left(\int_{V}{\left[N_{\delta }\right]}^{T}{\left[\dot{R}\right]}^{T}\left[R\right]\left[N_{\delta }\right]\rho_{o}dV\right){\left\{\dot{\delta }\right\}}_{L}+\left[\left[k\right]+\left[k_{v}\right]+\left(\int_{V}{\left[N_{\delta }\right]}^{T}\left({\left[\ddot{R}\right]}^{T}\left[R\right]\right)\left[N_{\delta }\right]\rho_{o}dV\right)\right]{\left\{\delta \right\}}_{L}\\ +\left[\left(\int_{V}{\left[N_{\delta }\right]}^{T}\rho_{o}dV\right){\left\{{\ddot{r}}_{O}\right\}}_{L}+\left(\int_{V}{\left[N_{\delta }\right]}^{T}{\left[R\right]}^{T}\left[\ddot{R}\right]{\left\{r\right\}}_{L}\rho_{o}dV\right)\right]-{\left\{q\right\}}_{L}^{T}-{\left\{q^{*}\right\}}_{L}^{T}-{\left\{q_{v}\right\}}^{T}=0 \end{array}$$

with the notation:

(53) $$\left[m\right]=\int_{V}{\left[N_{\delta }\right]}^{T}\left[N_{\delta }\right]\rho_{o}dV;\;\left[J\right]=\int_{V}{\left[N_{\varphi }\right]}^{T}\left[Y\right]\left[N_{\varphi }\right]\rho_{o}dV;\;\left[m_{\nu }\right]=\int_{V}{\left[N_{\nu }\right]}^{T}\left[N_{\nu }\right]\kappa \rho_{o}dV;$$

(54) $$\left[c\right]=2\int_{V}{\left[N_{\delta }\right]}^{T}{\left[\dot{R}\right]}^{T}\left[R\right]\left[N_{\delta }\right]\rho_{o}dV;\;\left[k(\varepsilon )+k(\omega^{2})\right]=\int_{V}{\left[N_{\delta }\right]}^{T}\left({\left[\ddot{R}\right]}^{T}\left[R\right]\right)\left[N_{\delta }\right]\rho_{o}dV;$$

(55) $$\left[m_{O}^{i}\right]=\int_{V}{\left[N_{\delta }\right]}^{T}\rho_{o}dV;\;\left\{q^{i}(\varepsilon )\right\}+\left\{q^{i}(\omega^{2})\right\}=\int_{V}{\left[N_{\delta }\right]}^{T}{\left[R\right]}^{T}\left[\ddot{R}\right]{\left\{r\right\}}_{L}\rho_{o}dV$$

This results in the final form of the motion equations:

(56) $$\begin{array}{c} (\left[m\right]+\left[J\right]+\left[m_{\nu }\right]){\left\{\ddot{\delta }\right\}}_{L}+\left[c\right]{\left\{\dot{\delta }\right\}}_{L}+\left(\left[k\right]+\left[k_{\nu }\right]+\left[k(\varepsilon )\right]+\left[k(\omega^{2})\right]\right){\left\{\delta \right\}}_{L}\\ ={\left\{q\right\}}_{L}^{T}+{\left\{q^{*}\right\}}_{L}^{T}+{\left\{q_{v}\right\}}^{T}-\left[m_{O}^{i}\right]{\left\{{\ddot{r}}_{O}\right\}}_{L}-\left\{q^{i}(\varepsilon )\right\}-\left\{q^{i}(\omega^{2})\right\}. \end{array}$$

The type of finite element chosen, the independent coordinates used, and the shape functions determine, in each case, the shape and content of the matrix coefficients in the equations.

## 5. Conclusions and Discussion

**Figure 1:**
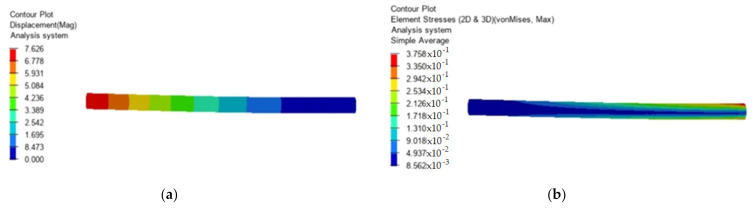
Material without voids. (**a**) Displacement (mm); (**b**) Stress (MPa).

**Figure 2:**
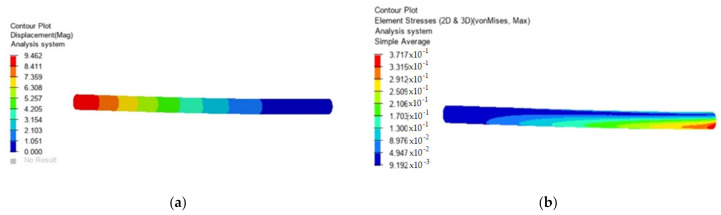
Material with voids (2%). (**a**) Displacement (mm); (**b**) stress (MPa).

To illustrate the method, the case of a bar was considered, fixed at one end and actuated at the other end with an axial force of 1000 N and a shear force of 1000 N. The length of the bar was considered to be 300 mm and its diameter 16 mm. The material of which the bar is made is a composite reinforced with carbon fibers. It was assumed that 2% voids would be found in the material. To determine the static response in this case, finite element methods were used, considering the development of this paper. Only the study for this bar request was carried out. The obtained results are presented in Figure 1 where the displacements and tensions are presented in the case of a material without voids and in Figure 2 in the case of a material that contains 2% voids.

The following conclusions can be drawn:

- The main phase in approaching MBS systems with elastic elements made of void materials is the writing of equations of motion. In this stage, thorough knowledge of the elastic properties of the worn material and of the constitutive laws is necessary. Once these equations are obtained, the other steps to be followed, namely, the assembly of the equations of motion and their solution, will be calculated according to the classical procedures currently used in common FEM software. To obtain these equations, a considerable amount of effort can be required, their shape depending both on the properties of the studied material but also on the chosen shape functions.
- The method used in the paper is Lagrangian formalism, which is used in most articles that study this issue. The use of this method has the advantage of a homogeneous writing and allows the automation of intermediate stages and the use of previously obtained results by other authors for parts of these equations. The major advantage of using this formalism is the frequent previous use and the existence of a rich experience in this use. The Lagrange method has a major advantage, namely, the formal use of well-known sizes in mechanics as kinetic energy, potential energy, and mechanical work, notions with which we are very accustomed and with which we operate easily. The use of other methods, although possible, has rarely been used by researchers [27,28,29,30,31,32,33,34,35,36,37]. Consideration concerning the future development of the method can be found in [55,56,57,58,59,60,61,62,63]. Another approach would be to use the Gibbs–Appell method, which seems to have the advantage of obtaining savings during modeling and calculation itself. However, this method has the disadvantage that researchers have to use a notion they are unfamiliar with, which is the energy of accelerations. A method consisting of applying Maggi’s equations can become useful when analyzing multibody systems in which the liaisons between the elements are nonholonomic. Hamiltonian formalism, another way of studying, can be advantageous from the point of view of a numerical calculation, finally obtaining a system of first-order differential equations. However, this method also has the disadvantage of an increased complexity of intermediate calculations. The last method we mentioned, that of Kane’s equations, is equivalent to Maggi’s formalism.
- In manufacturing, if a resin that has a high viscosity is used, voids in the material will most likely appear. This is because it is difficult for a resin with a high viscosity to penetrate and clog all of the empty spaces between the fibers. If the fiber concentration is very high, removing these voids is very difficult, if not impossible. It is obvious that often, in practical applications, we cannot remove them and we must take them into account when making calculations, as their presence can significantly change the mechanical properties.

## Data Availability

Not applicable.
